# Sentinel Lymph Node Biopsy: Is There a Role in Non-Melanoma Skin Cancer? A Systematic Review

**DOI:** 10.3390/cancers16244279

**Published:** 2024-12-23

**Authors:** Lorenzo Borgognoni, Pietro Susini, Gianni Gerlini, Paola Brandani, Vanni Giannotti, Serena Sestini

**Affiliations:** 1 Plastic and Reconstructive Surgery Unit, Regional Melanoma Referral Center and Melanoma & Skin Cancer Unit, Santa Maria Annunziata Hospital, 50012 Florence, Italy; gianni.gerlini@uslcentro.toscana.it (G.G.); paola.brandani@uslcentro.toscana.it (P.B.); vanni.giannotti@uslcentro.toscana.it (V.G.); serena.sestini@uslcentro.toscana.it (S.S.); 2 Plastic Surgery Unit, Department of Medicine, Surgery and Neuroscience, University of Siena, 53100 Siena, Italy; p.susini@student.unisi.it

**Keywords:** Non-Melanoma Skin Cancer and Sentinel Node Biopsy, Squamous Cell Carcinoma and Sentinel Node Biopsy, Merkel Carcinoma and Sentinel Node Biopsy, Porocarcinoma and Sentinel Node Biopsy

## Abstract

Background/Objectives: Sentinel Lymph Node Biopsy (SLNB) aims at identifying clinically occult nodal metastases. It is the standard staging procedure for patients with T1b to T4 primary cutaneous melanoma. Moreover, it is recommended whenever the risk of a positive SLNB is >5%, according to the National Comprehensive Cancer Network Melanoma guidelines. When considering Non-Melanoma Skin Cancer (NMSC), the SLNB could play a role in tumors that mainly spreads via lymphatics, but strong evidence is missing. In this paper, the hot topics and controversies are reviewed; Methods: A PRISMA systematic review was carried out on the PubMed (MEDLINE) library from 2004–2024, searching for studies on SLNB in NMSC; Results: Seventy articles and 6379 patients undergoing SLNB for Squamous Cell Carcinoma (SCC), Merkel Cell Carcinoma (MCC), and Porocarcinoma were included. Overall, the SLNB positivity rate in these NMSCs was 24.4%, with an SNLB detection rate of 97.6%. Specifically, the SLNB positivity rate was 12.3% for high-risk cutaneous SCC, 24.4% for anogenital SCC, 29.3% for MCC, and 30.6% for Porocarcinoma. Most papers concluded that SLNB is safe, feasible, and significant in these malignancies; Conclusions: SLNB should be discussed and offered to every patient with MCC, and it should be discussed and considered in “high risk” SCC and Porocarcinoma for staging and prognostic purposes, aiming to identify a subgroup of patients who may benefit the most from early treatments.

## Simple Summary

Sentinel Lymph Node Biopsy (SLNB) aims at the early detection of lymph node metastases. In the field of skin cancer, it is a standard staging procedure for patients with T1b to T4 primary cutaneous melanoma. When considering Non-Melanoma Skin Cancer (NMSC), the SNLB should be rationally considered in tumors with a typical lymphatic spread, including Squamous Cell Carcinoma, Merkel Cell Carcinoma, and Porocarcinoma. However, the SLNB-NMSC criteria, thresholds, and guidelines are currently missing. Hereby, the role of SNLB in NMSC is reviewed.

## 1. Introduction

Sentinel Lymph Node Biopsy (SLNB) is as a surgical procedure aimed at identifying clinically occult regional metastases of the lymph nodes. Among skin tumors, it represents the standard staging procedure for patients with T1b, T2, T3, and T4 primary cutaneous melanoma [1]. In these patients, the SLNB positivity rate is approximately 20%, ranging from 5–40%, depending on the primary tumor [2]. SLNB is also recommended whenever the risk of a positive SLNB is >5%, according to the National Comprehensive Cancer Network (NCCN) Melanoma guidelines [3]. Therefore, SLNB criteria are also identified for thin melanomas, such as in the case of ulceration or mitosis ≥2 [4].

SLNB, first introduced in melanoma and breast cancer, has been applied to other tumors, i.e., thyroid, endometrium, oral cavity, and prostate [5,6,7,8,9,10,11]. In the field of Non-Melanoma Skin Cancer (NMSC), the SLNB could be indicated in those malignancies with a prevalent lymphatic spread, including Squamous Cell Carcinoma (SCC), Merkel Cell Carcinoma (MCC), and rare adnexal tumors such as Porocarcinoma. However, SLNB criteria, thresholds, and guidelines are currently missing.

SCC is the paradigm of a tumor with a usually orderly and stepwise progression, even more so than melanoma, with metastases occurring primarily as local disease, extending to regional lymph nodes and then, subsequently, and in a minority of patients, to distant sites [12]. Most SCCs have a favorable prognosis, but 5% of patients may develop metastasis, significantly impacting prognosis. Moreover, “high risk” SCCs have been described, such as in [12]. These present a greater probability of lymph node metastatic diffusion, up to 15–20%. Patients with lymph node metastases have an inauspicious prognosis, with 5-year survival rates of 26–34% [12]. However, early treatment could be beneficial. Indeed, when a single node is involved and extracapsular spread has not occurred, 5-year survival rates increase up to 75% [13]. Thus, SLNB in high-risk SCC could be appropriate, aiming for the early identification of clinical occult metastasis of the lymph nodes.

MCC is an aggressive neuroendocrine malignancy. Lymph node metastases occur in up to 20% of MCC patients, and survival rates are low, at 65%, 40%, and 18% for local disease, lymph node, or distant metastases, respectively [14].

Porocarcinoma is an adnexal tumor that arises from the intraepidermal portion of the eccrine glands’ ducts [15,16]. It is a biologically aggressive tumor, with lymph node and systemic metastasis rates of 20% and 10%, respectively [15,16]. It is also poorly responsive to chemo and radio therapies [15,16]. Porocarcinoma initially spreads through the lymphatic route [17], and lymph node metastases increase mortality up to 65% [15,16].

When considering the aforementioned rates, the SNLB should be rationally considered, aiming at the early detection of lymph node metastases in skin tumors with a typical lymphatic spread. With the present paper, we review the hot topics and controversies regarding the role of SLNB in NMSC, aiming to improve the tumor staging and management.

## 2. Materials and Methods

### 2.1. The Data Sources and Search Strategy

According to the PRISMA statement for Systematic Reviews [18], a comprehensive literature search was conducted on the PubMed (MEDLINE) library from August 2004–August 2024, using the terms ‘‘(Non Melanoma Skin Cancer) OR (Squamous Cell Carcinoma) OR (Merkel Carcinoma) OR (Porocarcinoma) AND (Sentinel Node Biopsy)”. An extensive list of terms to describe the target population based on the PICO acronym was formulated, as follows:

P (population)—Non-Melanoma Skin Cancer;

I (intervention)—Sentinel Node Biopsy;

C (comparator)—control group, absence of Sentinel Node Biopsy, lymphadenectomy, and medical treatment;

O (outcomes)—SLNB positivity rate, SNLB detection rate, and assessment of any SLNB beneficial roles.

This systematic review was registered in the International Prospective Register of Systematic Reviews (PROSPERO), ID: CRD42024588668.

### 2.2. Study Selection

The inclusion criteria were original studies (observational studies or randomized controlled trials) reporting SLNB in skin SCC, MCC, and Porocarcinoma. Various aspects of SLNB in the setting of NMSC were focused, including the SNLB positivity rate and SNLB clinical utility. Since we evaluated the positivity rate, studies that specifically enrolled only SLNB-positive or SLNB-negative cases were excluded.

For SCC, our focus was cutaneous SCC (cSCC), including head and neck cutaneous lesions. Articles on head and neck mucosal SCC, as well as oropharyngeal and laryngeal SCC, were excluded, considering the different nature of these malignancies. Studies on genital SCC (penile, vulvar, and anal–perianal) were enrolled, considering the typically mixed cutaneous–mucosal involvement. Studies were also excluded if they were animal studies, review articles, or meta-analyses, books and documents, case reports, letters to the editor, and papers not written in English. Case series were included if they reported a minimum of two cases undergoing SLNB.

The literature search was performed by one independent reviewer (P.S.). Following the title and abstract screening, we established whether publications met the selection criteria. Furthermore, when the title and abstract screening alone was unclear, the full text was reviewed and compared to the selection criteria. The bibliographical references were also screened. The included articles were then subjected to a full-text review and tested with the selection criteria. After the study selection, data extraction, and critical appraisal, the collected data were brought to the attention of the senior author (L.B.) for final approval and possible dispute resolution. Accordingly, the selected papers were re-examined and finally included to present the information in this review.

### 2.3. Data Extraction and Analysis

Data were extracted on patient demographics, lesion characteristics, and SLNB procedures. Demographics included age and gender. Lesions were assessed for hystotypes and anatomical regions, including face and neck, upper limbs, lower limbs, trunk, vulvar, penile, and anal–perianal lesions.

Outcomes of interest included the SLNB positivity rate. The latter was calculated as the number of patients with NMSC undergoing SLNB with at least 1 positive lymph node/all patients undergoing SLNB. Additionally, we calculated the SNLB detection rate, defined as the number of SNLB procedures with an identification of at least 1 lymph node/all procedures, aiming to assess the feasibility of the procedure. Finally, we distinguished the included papers into studies that considered the SLNB safe, feasible, and significant, and studies that considered SLNB to be poorly significative, based on the authors’ personal interpretation of their conclusions.

Separate outcomes were obtained for SCC, MCC, and Porocarcinoma. Moreover, subanalysis was conducted on SCC, further distinguishing the data for cSCC and anogenital SCC.

SLNB procedural technicalities and complication rates were not a parameter of interest and were not investigated. Furthermore, the oncological outcomes and survival rates were not an endpoint and were not considered in this review.

## 3. Results

**Figure 1:**
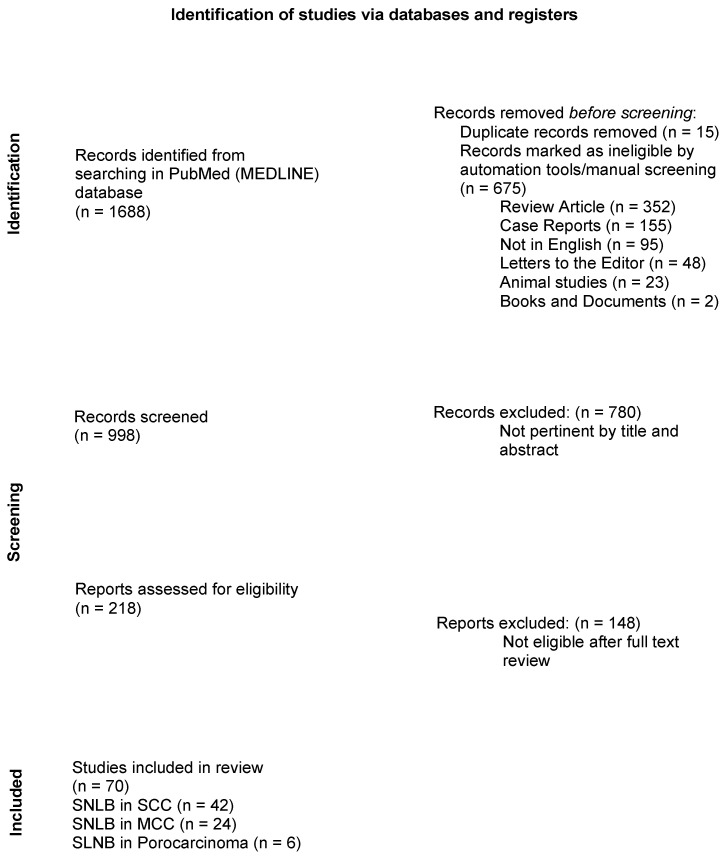
PRISMA 2020 flow diagram summarizing research results.

Based on the established keywords, the primary research yielded a total of 1688 articles. These were compared to the selection criteria. By using PubMed’s automatic search tools and manual screening, 352 reviews and meta-analyses, 155 case reports, 95 articles not written in English, 48 letters to the editor, 23 animal studies, and 2 books/documents were excluded. Fifteen duplicates were also excluded. The remaining articles were assessed for relevance based on their titles and abstracts; as a result, 218 potentially eligible original articles were selected and fully reviewed. Of these, 148 articles that were not relevant to the aim of this study were excluded. Finally, 70 articles met the selection criteria and were included in this review (Figure 1). These were classified into studies reporting SLNB for SCC (n. 42, Table 1), studies on SLNB for MCC (n. 24, Table 2), and studies on SLNB for Porocarcinoma (n. 6, Table 3).

### Demographics, Lesion Characteristics, and Outcomes

Patient demographics, lesion characteristics, and outcomes are summarized in Table 4. Our analysis includes 70 studies reporting data on 6379 patients who underwent SLNB for NMSC. Data on gender was available for 5837 of the patients (91.5%). Of these, 2654 (45.5%) were females and 3183 (54.5%) were males. The average age was 65 years old (average age range: 59–74 years). The anatomical site of the primary lesion was available for 4478/6379 (70.2%). Of these, 33.7% (1508/4478) was the face and neck, 3.3% (147/4478) was the upper limb, 4.0% (179/4478) was the lower limb, 5.1% (229/4478) was the trunk, 30.0% (1343/4478) were vulvar, 19.2% (859/4478) were penile, and 4.8% (213/4478) were anal.

Overall, the calculated SLNB positivity rate was of 24.4% (1557/6379). Data on the SNLB detection rate were available for 5050 patients (79.0%). In this cohort, the SLNB detection rate was 97.6% (4930/5050). Among the 70 included articles, 63/70 studies (90.0%) described the SLNB as a safe, feasible, and significative procedure, while 7/70 (10.0%) concluded that SLNB is poorly significative.

For SCC, 42 studies reported data on 3546 SCC patients undergoing SLNB (55.6% of NMSC patients). Data on gender were available for 3349 of the patients (94.4%). Of these, 1679 (50.1%) were females and 1670 (49.9%) were males, and the average age was 62 years old (59–73 years). The anatomical site was available for 3372/3546 (95.0%). Of these, 24.7% (832/3372) was the face and neck, 1.4% (48/3372) was the upper limb, 1.8% (59/3372) was the lower limb, 0.4% (13/3372) was the trunk, 39.9% (1346/3372) were vulvar, 25.5% (859/3372) were penile, and 6.3% (213/3372) were anal.

The calculated SLNB positivity rate was 20.5% (726/3546). Data on the SNLB detection rate were available for 2946 patients (83.0%). The SLNB detection rate was 96.4% (2841/2946). Among the 42 articles, 37/42 studies (88.1%) described SLNB as safe, feasible, and significative, while 5/42 (11.9%) concluded that SLNB is poorly significative.

Data on SCC were further distinguished into studies of cSCC and SCC of the anogenital region. Among cSCC, we also detailed results of the skin SCC of the face and neck. Separate outcomes for trunk and extremities SCC were not obtained, given the lack of data from the included papers.

- The cSCC subgroup included 26 studies and 1143 patients (32.2% of all SCC), of which data on gender were available for 964/1143 of the patients (87.3%). Of these, 24.8% (239/964) were females, 725/964 (75.2%) were males, and the average age was 65 (60–74). This group included 88.1% of face and neck SCC, 4.5% of upper limb SCC, 5.8% of lower limb SCC, and 1.6% of trunk SCC. The calculated SLNB positivity rate was 12.3% (141/1143). Data on SNLB detection rate were available for 958 patients (83.8%). In this cohort, the SLNB detection rate was 93.1% (892/958). Among the 26 articles, 21/26 studies (80.8%) described the SLNB for cSCC as safe, feasible, and significative, while 5/26 (19.2%) concluded that SLNB is poorly significative.
- For the face and neck cSCC subgroup, 10 studies and 540 SCC patients were reported (15.2% of all SCC and 47.2% of all cSCC). Data on gender were available for 458/540 of the patients (84.8%). Of these, 22.7% (104/458) were females, 354/458 (77.3%) were males, and the average age was 77 (60–73). Data on the SNLB detection rate were available for 475 patients (88.0%). The SLNB detection rate was 97.9% (465/475) and the SLNB positivity rate was 15.9% (86/540). All 10 articles concluded that SLNB for face and neck SCC is safe, feasible, and significative. Since we focused on skin SCC, oropharyngeal and laryngeal (mucosal) SCC were excluded. Our data refer to face and neck cSCC, including the lip vermilion, rather than the head and neck in general. In fact, tumors of the lip vermilion are not considered among the cancers of the oral cavity in the “NCCN guidelines for head and neck cancers” [88]. Contrariwise, the latter includes tumors of the mucosa of the lip and the oral cavity.
- For anogenital SCC, 16 studies and 2403/3546 SCC patients (67.7%) were reported, with an average age of 63 (57–67). This group comprised 55.6% (1337/2403) vulvar SCC, 35.8% (859/2403) penile SCC, and 8.6% (207/2403) anal–perianal SCC. The detection rate was 98.0% (1946/1985). Specifically, the rates were 96.3% (885/919), 99.5% (855/859), and 99.5% (206/207) for vulvar, penile, and anal SCC, respectively. The SLNB positivity rate was 24.4% (585/2403). Specifically, it was 25.1% (335/1337), 23.5% (202/859), and 23.1% (48/207) for vulvar, penile, and anal SCC, respectively. All 16 articles concluded that SLNB for anogenital SCC is safe, feasible, and significative.

For MCC, 24 studies reported data on 2761 MCC patients undergoing SLNB (43.3% of NMSC patients). Data on gender were available for 2473/2761 of the patients (89.6%). Of these, there were 971/2473 (39.3%) females and 1502 (60.7%) males, and the average age was 70 years (69–73). The anatomical site was available for 1084/2761 (39.3%). Of these, 61.5% (667/1084) was the face and neck, 8.9% (96/1084) was the upper limb, 10.2% (111/1084) was the lower limb, and 19.4% (210/1084) was the trunk.

The SLNB positivity rate was 29.3% (809/2761). Among the 24 articles, 23/24 studies (96.8%) described the SLNB as safe, feasible, and significative, while 1/24 (4.2%) concluded that SLNB is poorly significative.

For Porocarcinoma, six studies reported data on 72 patients undergoing SLNB. Data on gender were available for 18/72 of the patients (25.0%). Of these, 7/18 (38.9%) were females and 11/18 were (61.1%) males, with an average age of 63 years (59–69). The anatomical site was available for all 18 lesions, of which 11.1% (2/18) were the face and neck, 16.7% (3/18) were the upper limb, 50.0% (9/18) were the lower limb, and 22.2% (4/18) were the trunk.

The SLNB positivity rate was 30.6% (22/72). Among the articles, 5/6 studies (83.3%) described SLNB as safe, feasible, and significative, while 1/6 (16.7%) concluded that SLNB is poorly significative.

## 4. Discussion

The present review analyzed 70 articles reporting data on 6379 patients who underwent SLNB for NMSC, SCC, MCC, and Porocarcinoma. We found an SLNB positivity rate of 24.4%, with an SNLB detection rate of 97.6%. Most papers concluded that the SLNB is safe, feasible, and significant in these skin tumors.

### 4.1. Squamous Cell Carcinoma

SCC is an aggressive tumor characterized by a stepwise progression from local disease to regional lymph nodes, and then, eventually, to distant sites [12]. High-risk SCCs have been described, with a 15–20% probability of metastatic spread [11]. However, the definition of “high risk” SCC is currently a topic of debate, and standard criteria are missing [89]. The NCCN guidelines include various factors, while the American Joint Committee on Cancer (AJCC) is more selective, and the Brigham and Women’s Hospital (BWH) classification is the most selective, as it only considers the following criteria: tumor size >2 cm, poorly differentiated, perineural invasion, and bone invasion [3] (Table 5).

An important contribution to the definition of high-risk SCC comes from the meta-analysis by Thompson et al. [90] in 2016, reporting data on 17,248 patients from 36 studies. The authors concluded that the statistically significative factors for SCC metastasis were size >20 mm; Breslow thickness >6 mm; poorly differentiated; perineural invasion; extension beyond subcutaneous fat; anatomical region of the lip, ear, and temporal region; and immunodepression. However, to date, the BWH probably remains the most objective classification; based on the results of the analysis of a series of 1800 patients, it concludes that the probability of SCC metastasis is influenced by the presence of defined risk factors (Table 5) [91]. Such probability increases when multiple factors occur simultaneously. Consistently, various authors have attempted to correlate the risk of nodal metastasis depending on the characteristics of the primary SCC. Among these, Braum et al. [12] demonstrated that lymph node metastases occur in 12% of cases of cancer >2 cm and with the concomitant presence of one additional “high risk” factor, therefore justifying the SLNB. Such risk rises to 22–67%, according to the presence of additional risk factors, such as perineural or deep invasion and poor differentiation [12].

These data are necessary for a rational debate on the use of the SLNB in SCC, and future research is warranted to better clarify the definition of “high risk” SCC (and therefore the criteria for SLNB in SCC), possibly referring to the statistical analysis of the greatest possible number of case studies, in an evidence-based medicine perspective.

#### The Role of SLNB in High-Risk SCC

In the present review, we present data on 3546 SCC patients undergoing SLNB. Overall, our findings include the calculated SLNB positivity rate in SCC of 20.5% (726/3546) of the patients. Distinguishing cSCC (face and neck, trunk, and extremities) from the SCC of the anogenital region, the SLNB positivity rates were 12.3% (15.9% for face and neck SCC) and 24.4%, respectively, which are significantly supportive towards the use of SLNB in SCC patients.

The SNLB detection rate was 96.4%, attesting to the feasibility of the procedure. Among the 42 articles, 37/42 studies (88.0%) described SLNB as safe, feasible, and significative, while 5/42 (11.9%) concluded that SLNB is poorly significative.

Our data agree with the recent literature. In 2017, Ahadiat et al. [92] published a review article of 14 studies on SLNB for 260 high-risk SCC cases. The authors reported a 14% positivity rate for SLNB, promoting the use of SLNB in these patients. This percentage is similar to the percentage of 12.3% found in our review on 26 studies on cSCC, and to the percentage of 15.9% in the subgroup of 10 studies referring only to face and neck SCC. The SLNB detection rates were 93.1% and 97.9% for cSCC and face and neck SCC, respectively. Interestingly, the detection rate of face and neck SCC is higher than the all-sites cSCC detection rate, and all 10 articles on face and neck SCC concluded that SLNB is safe, feasible, and significative.

In the present review we also reported data on vulvar, penile, and anal SCC. Tumors arising in these areas often have a mixed cutaneous–mucosal involvement, and they are often associated with Human Papillomavirus (HPV) infection [93]. The detection rates were 96.3%, 99.5%, and 99.5%, for vulvar, penile, and anal SCC, respectively. All studies concluded that SLNB was safe, feasible, and significant, as reported by previous papers [94,95]. The SLNB positivity rates were 25.1%, 23.5%, and 23.2%, for vulvar, penile, and anal SCC, respectively. This significantly higher positivity rate of the sentinel lymph nodes in these tumor subsets compared to cSCC might reflect a different etiology and the possibility that some mucosal SCCs are included in the studies, especially regarding the anal region. In particular, the papers investigating SLNB in the anal region often included SCCs of the anal canal. However, the studies concluded that the procedure was also feasible in anal SCCs scheduled for chemoradiation therapy, and it is useful in order to select patients with negative SLNB that could avoid irradiation of the inguinal lymph nodes. After diagnostic biopsy, the procedure requires preoperative lymphoscintigraphy with an injection of the tracer around the anal lesion, selection of patients for SLNB who have drainage to the inguinal lymph nodes, identification of the sentinel node with a gamma probe, and excision [22,44,49,96].

Remarkably, Gore et al. [36] reported data on SLNB in SCC and survival rates. The authors found that patients with SSC and positive SLNB have a significantly lower survival rate compared to SNLB-negative patients [36], as observed in melanoma patients. Therefore, the SLNB allows us to correctly stage SCC patients and identify a group of patients with worse prognosis who can benefit the most from early treatment and strict follow-up.

It is noteworthy that all 42 articles reviewed in this paper enrolled “high risk” SCC cases. Consequently, the calculated outcomes in terms of the SNLB positivity rate and SNLB clinical utility refer to “high-risk” SCC, rather than to SCCs in general. The included papers defined “high risk SCC”, referring to different criteria, including the NCCN, the AJCC, or BWH criteria for high-risk SCC, potentially influencing a true comparison. This possible bias represents a limitation of the study, but it was still accepted, considering the absence of common consent in the scientific community for “high risk” SCCs. The comparison between SLNB positivity rates for “high-risk” and “non-high-risk” SCC cases was not possible due to inadequate data and the lack of a univocal definition of “high-risk” SCC. Indeed, the study of prognostic factors for SCC should become a topic of future research.

However, whatever the criteria used to define high-risk SCC cases (NCCN, AJCC, BWH), the sentinel node positivity reported in the literature is high, being 12.3% for cSCC and 24.3% for anogenital SCC. Therefore, taking into account the positivity threshold criterion adopted to recommend SLNB in melanoma, which is >5% [3,4], SLNB should be discussed and offered to all high-risk SCC patients—whatever the criteria used—for staging and prognostic purposes, in order to identify a subgroup of patients with occult lymph node metastases who would be otherwise incorrectly under-staged, and who may benefit the most from early surgical, and possibly medical, treatments. A clear and homogeneous definition of “high risk” SCC remains to be established.

### 4.2. Merkel Cell Carcinoma

MCC is an aggressive tumor with a large proportion of lymph node metastases. In 2010, Lemos et al. [97] reported 5823 MCC patients from the National Cancer Database of Washington, demonstrating that pathologic nodal evaluation improves prognostic accuracy in MCC. Comparing clinical and pathological nodal staging (cN vs. pN staging) in patients with MCC, cN+ patients have a survival rate of 26%, while pN+ patients identified by SLNB have a survival rate of 42% [97], suggesting a possible benefit from early lymph node metastasis detection and treatment. In addition, pN patients, i.e., those who are SLNB-negative, have a survival rate of 76%, which is 15% higher than the rate for cN- patients [97], which is possibly explained by the fact that the latter group of patients who have not undergone SLNB includes patients with clinically occult lymph node metastases in the lymph nodes, with a worse prognosis. This is in agreement with the data and survival curves previously observed in melanoma, where SLNB allows for more appropriate staging [98].

#### The Role of SLNB in Merkel Cell Carcinoma

In the present literature, we identified 24 studies discussing the role of SLNB in MCC. We included 2761 patients undergoing SNLB. Among the 24 included articles, the SNLB detection rate was 99.3%, with 23/24 studies (96.8%) describing SLNB as safe, feasible, and significative. The SNLB positivity rate was 29.3%, thus stressing the importance of performing SNLB in these patients.

Our data agree with the present literature. Sims et al. [66] reported an SLNB positivity rate of 25–30% in MCC patients. Unlike melanoma, no correlation has been identified between histological features and sentinel positivity [99]. Tumor size, on the other hand, correlates with the positivity of the sentinel test [66,100]. However, even in small tumors (<0.5 cm), the SLNB is positive in 15% of cases [66,100]. Furthermore, the meta-analysis by Sadeghi et al. [101] demonstrated that SNLB is a significant prognostic factor in patients with MCC, thus influencing survival rates. Patients with a positive sentinel node have a four-times higher probability of death compared to SNLB-negative patients [101]. Consistently, the AJCC 8th ed. Merkel staging classification considers SLNB for lymph nodes’ staging. The staging includes the pN1 category of patients with clinically occult lymph node metastasis, necessarily identified by SNLB.

Overall, SLNB is recommended for the appropriate management of MCC in clinically N0 patients [102]. It allows for the correct staging of the patient according to the AJCC 8th ed. Moreover, it enables the identification of a subgroup of high-risk patients who would be otherwise incorrectly under-staged, and who may benefit the most from early treatment.

### 4.3. Porocarcinoma

Porocarcinoma is a rare adnexal tumor. Metastases of the lymph nodes are reported in 20% of cases, while distant sites are affected in 10% [15,16]. To date, thickness ≥ 7 mm and mitosis > 14/mm^2^ are considered unfavorable prognostic factors, predictive of lymph node metastasis, together with lymphovascular invasion and a poorly differentiated tumor [15,103]. However, strong evidence is missing, mainly due to its rarity.

#### The Role of SNLB in Porocarcinoma

We report six studies and 72 Porocarcinoma patients undergoing node biopsy, with an SNLB positivity rate of 30.6% (22/72) and an SNLB detection rate of 100%. Among the 6 papers, 5/6 studies (83.3% %) described SLNB as safe, feasible, and significant, while the 2021 paper by Goyal et al. [84] concluded that SNLB was poorly significative. Notably, their study was mainly focused on adnexal malignancies, reporting 7591 cases (and only 50 Porocarcinoma). Reasonably, their conclusions were mainly directed to all adnexal malignancies, rather than specifically in Porocarcinoma. Moreover, no clear information was provided on the presence of risk factors for nodal metastasis, and therefore it is not clear if the included cancers were high-risk or not.

In accordance with our data, in 2018, Nazemi et al. [104] published a review on Porocarcinoma that included 153 studies and 206 patients, mostly case reports, reporting 16 SLNB procedures for high-risk Porocarcinoma and a 14/16 (87.5%) of SNLB positivity rate, therefore promoting the SNLB. In addition, Tsunoda et al. [17] reported eight patients without clinical or instrumental signs of lymph node metastases who underwent SNLB. Three were positive and, therefore, lymphadenectomy was performed. All three were alive at an average follow-up of 30 months [17].

Overall, SNLB may have a role in Porocarcinoma, given that this cancer mainly spreads via the lymphatic system, and early detection of clinical occult metastasis may be indicative for additional treatments. However, the indications remain to be verified, particularly in non-high-risk Porocarcinoma. These latter criteria are yet to be confirmed and validated, and future studies are warranted in larger case series.

### 4.4. Study Limitations

A limitation of this systematic review is that the papers included for the analysis on the possible role of SLNB in high-risk SCC referred to the “high risk” definition according to the AJCC, BWH, NCCN, which used different criteria to identify “high risk” SCC. However, there is no univocal definition in the literature for high-risk SCCs, and the percentage of positive SNLBs is >10%, no matter which definition was used. Another limitation of this review is that it only includes the PubMed (Medline) library, while other databases were not searched. We selected articles of SNLB in NMSC and assessed the SNLB positivity rate, the SNLB detection rate, and SNLB utility (safe, feasible, and significant vs. poorly significative). The latter criteria are non-recognized, non-validated, and based on the authors’ personal interpretation of the included articles’ conclusions. Moreover, we only selected studies reporting SLNB procedures. Therefore, it is logical that most authors consider SLNB to be a useful procedure, otherwise they would not perform it. Furthermore, we have evaluated the SNLB for SCC, MCC, and Porocarcinoma, while other NMSC subtypes were not considered. Future studies are warranted.

## 5. Conclusions

SLNB is positive in 12.3% of high-risk cSCCs and in 24.4% of SCCs of anogenital region. Even if the definition of “high risk” SCC is unclear in the literature, the sentinel node is positive in >10% of the “high risk” SCCs, no matter which classification was used. SLNB is positive in 15–50% of MCCs, and provides accurate staging and strong prognostic information regarding the overall survival and disease-specific survival. SLNB is positive in 30.6% of Porocarcinoma, a radio–chemo resistant malignancy that mainly spreads via the lymphatics. Due to its rarity, the role of SLNB and possible high-risk features remain to be clarified in large case series.

As in the case of melanoma, SLNB could be discussed and offered in high-risk SCC, MCC, possibly Porocarcinoma cases, as well as other NMSCs that mainly spread through the lymphatic route, when the probability of the positive sentinel node is >5%, in order to correctly stage the patient, have significant prognostic information, perform early surgery, radiotherapy, and medical treatment—which are increasingly important with the advent of new medical therapies in NMSC—and to carry out the appropriate follow-up. Although adjuvant therapies are not approved in NMSC, the excellent results obtained with the new therapies in advanced and metastatic SCC and MCC could also suggest a possible role for these drugs in the adjuvant setting in SLNB-positive patients. Similar to melanoma, SLNB can identify patients with lymph node metastases early, and thus they could be introduced to systemic therapies for NMSC [105,106].

Finally, the SNLB in NMSCs could allow the in vivo study of tumor progression mechanisms and metastatic spread. In particular, SCC could represent the in vivo model of the incubator hypothesis [107], in which the sentinel node is a sort of incubator, where the micrometastatic deposit can remain for a long time, grow slowly, and, subsequently, even after a long time, migrate to other distant organs. On the other hand, MCC may represent the in vivo model of the marker hypothesis [107], in which a positive sentinel node can represent a marker of a biologically aggressive tumor, in which metastatic spread may have occurred from the beginning simultaneously, by the blood and lymphatic route, which is potentially indicative and useful for early additional therapies.

## Figures and Tables

**Table 1:** Clinical evidence for the role of SLNB in SCC.

Reference	N. Patients	Females	Males	Mean Age	Body Region	SLNB Positivity Rate Per Carcinoma	Study Field	Outcomes
Tejera-Vaquerizo et al. 2024 [19]	70	NA	NA	NA	NA	11/70 (15.7%)	High-risk cSCC	High-risk cSCC disease-specific survival is improved by SNLB in immunocompetent but not in immunosuppressed patients.
Janković et al. 2021 [20]	64	24	40	71 (58–84)	Face and neck: 64	14/64 (21.9%)	Face and neck high-risk SCC	In these patient SLNB predicts potential metastatic sites.
Ilmonen et al. 2021 [21]	63	22	41	71 (40–91)	Face and neck: 63	4/63 (6.4%)	Face and neck high-risk cSCC	Apparently, SLNB has no prognostic value for identifying recurrent face and neck high-risk cSCC.
De Nardi et al. 2021 [22]	123	76	47	60 (57–72)	Anal: 123	28/123 (22.8%)	Anal cSCC	Locoregional control and prognosis are not compromised by a negative SLNB.
Kofler et al. 2021 [23]	150	24	126	71	Face and neck: 131 Extremities: 29	6/150 (4.0%)	High-thickness (>5 mm) cSCC	No strong evidence of beneficial role.
Froeding et al. 2020 [24]	286	286	0	67 (32–97)	Vulvar: 286	96/286 (33.6%)	Vulvar cSCC	SLNB-negative vulvar cSCC patients have lower nodal recurrence and good disease-specific survival.
Hermann et al. 2020 [25]	304	304	0	NA	Vulvar: 304	34/304 (11.2%)	Vulvar cSCC.	SLNB is related to increased periperative outcomes but has no impact on overall survival.
Wu et al. 2020 [26]	83	15	68	69 (17–89)	Face and neck: 83	6/83 (7.2%)	Face and neck high-risk cSCC	SLNB has high negative predictive value (95%–100%). Recurrence risk factors include the following: previous recurrence, tumors arising in area of chronic inflammation, and immunosuppression.
Broach et al. 2020 [27]	114	114	0	63 (19–98)	Vulvar: 114	25/114 (21.9%)	Vulvar cSCC	SLNB for vulvar cSCC is oncologically effective. Indocyanine green is helpful for SLNB.
Chabrillac et al. 2019 [28]	74	9	65	72 (39–92)	Face and neck: 68 Extremities and trunk: 7	8/74 (10.8%)	cSCC	Tumor size and poor tumor differentiation are statistically significative risk factors for positive SLNB.
Mooney et al. 2019 [29]	104	14	90	65 (27–90)	Face and neck: 104	10/104 (9.6%)	Face and neck cSCC	SLNB for high-risk face and neck cSCC, SNB is a safe and feasible staging strategy.
Lhote et al. 2018 [30]	37	7	30	72 (65–78)	NA	1/37 (2.7%)	cSSC	SLNB does not affect relapse-free and overall survival rates; thus, it should not be mandatory in the management of cSSC.
Lonergan et al. 2017 [31]	11	0	11	63 (39–78)	Penile: 11	1/11 (9.1%)	Penile cSSC	SLNB is a safe and feasible staging technique.
Maruyama et al. 2017 [32]	49	18	31	66 (30–86)	Face and neck: 11 Upper limbs: 11 Lower limbs: 20 Trunk: 1 Anal: 6	9/49 (18.4%)	cSCC	SLNB does not affect metastasis-free and disease-specific survival rates, regardless of T staging.
Sollamo et al. 2016 [33]	26	10	16	71 (40–83)	Face and neck: 26	3/26 (11.5%)	Face and neck cSCC (lip)	SLNB is a safe and feasible staging technique for lip cSCC. Tumor thickness and size (>20 mm) correlated to higher risk of positive SLNB.
Durham et al. 2016 [34]	53	9	44	73 (47–90)	Face and neck: 53	6/53 (11.3%)	Face and neck cSCC	SLNB is a safe and feasible staging technique for face and neck cSCC. NCCN guidelines help identify patients at risk for positive SLNB.
Samsanavičius et al. 2016 [35]	51	34	17	74 (48–92)	Face and neck: 33 Upper limbs: 3 Lower limbs: 9 Trunk: 6	0/51 (0%)	cSCC	SLNB for cSCC correlates with prognosis and disease progression.
Gore et al. 2016 [36]	57	10	47	67 (29–90)	Face and neck: 57	8/57 (14.0%)	Face and neck cSCC	SLNB is a safe and feasible staging technique. (14% positive SLNB in face and neck high-risk SCC).
Dimopoulos et al. 2015 [37]	151	0	151	62 (35–89)	Penile: 151	37/151 (24.5%)	Penile cSSC	SLNB is a safe and feasible staging technique for penile cSCC. The 1-day protocol has a lower false-negative rate than the 2-day protocol.
Krediet et al. 2015 [38]	17	NA	NA	NA	NA	2/17 (11.8%)	cSCC	Negative SLNB patients presenting tumor thickness > 4 mm or with recurrent disease may develop metastases within the first 2 years and require close monitoring despite the SLNB result.
Takahashi et al. 2014 [39]	26	15	11	70 (47–88)	Face and neck: 5 Upper limbs: 5 Lower limbs: 7 Trunk: 2 Genitals: N/A	6/26 (23.1%)	cSCC	SLNB is a safe and feasible staging technique for cSCC. SLNB should be considered for cSCC >2 mm and strictly indicated for cSCC >5 mm in thickness.
Fukushima et al. 2014 [40]	54	17	37	69 (20–87)	Face and neck: 25 Upper limbs: 13 Lower limbs: 12 Trunk: 2 Genitals: N/A	4/54 (7.4%)	cSCC	SLNB is a safe and feasible staging technique for cSCC. Positive SLNB rates are comparable to that of melanoma.
Woelber et al. 2013 [41]	106	106	0	57 (20–87)	Vulvar: 106	33/106 (31.1%)	Vulvar cSCC	Delayed SLNB for vulvar cSCC is a safe, feasible, and significative staging technique.
Lam et al. 2013 [42]	264	0	264	66	Penile: 264	59/264 (22.4%)	Penile cSCC	SLNB for penile cSCC is a safe, feasible, and significative staging technique.
Kirrander et al. 2013 [43]	58	0	58	60 (37–84)	Penile: 58	11/58 (19.0%)	Penile cSCC	SLNB for penile cSCC is a safe, feasible, and significative staging technique.
Mistrangelo et al. 2013 [44]	63	24	39	59 (32–82)	Anal: 63	13/63 (20.6%)	Anal cSCC	SLNB for anal cSCC is a safe, feasible, and significative staging technique. It should be considered as a standard of care.
Matthey-Giè et al. 2013 [45]	8	3	5	62 (33–92)	Upper limbs: 3 Lower limbs: 4 Vulvar: 1	1/8 (12.5%)	cSCC	SLNB for cSCC is a safe, feasible, and significative staging technique.
Levenback et al. 2012 [46]	452	452	0	NA	Vulvar: 452	132/452 (29.2%)	cSCC	SLNB is a reasonable alternative to inguinal femoral lymphadenectomy for the selected vulvar cSCC.
Kwon et al. 2011 [47]	5	1	5	72 (51–89)	Face and neck: 2 Upper limbs: 2 Trunk: 1	0/5 (0.0%)	cSCC	SLNB for high-risk cSCC is a safe and feasible staging technique. Face and neck SCC present a lower false-negative rate and a higher negative predictive value compared to cSCCs located elsewhere.
Rastrelli et al. 2010 [48]	20	4	16	72 (49–90)	Face and neck: 11 Extremities: 8 Trunk: 1	1/20 (5.0%)	cSCC	SLNB for cSCC is a safe, feasible, and significative staging technique.
De Jon et al. 2010 [49]	21	NA	NA	NA	Anal: 21	7/21 (33.3%)	Anal cSCC	SLNB for anal cSCC is a safe, feasible, and significative staging technique. It also helps in identifying patients requiring inguinal irradiation.
Crosbie et al. 2010 [50]	32	32	0	67 (34–94)	Vulvar: 32	6/32 (18.8%)	Vulvar cSCC	SLNB for vulvar cSCC is a safe and feasible staging technique.
Achimas-Cadariu et al. 2009 [51]	43	43	0	66 (34–93)	Vulvar: 46	9/43 (20.9%)	Vulvar cSCC	SLNB for vulvar cSCC is a safe and feasible staging technique.
Leijte et al. 2009 [52]	323	0	323	64 (33–96)	Penile: 323	79/323 (24.5%)	Penile cSCC	SLNB for penile cSCC is a safe and feasible staging technique.
Jensen et al. 2008 [53]	52	0	52	62 (40–76)	Penile: 52	15/52 (28.9%)	Penile cSCC	SLNB for penile cSCC is a safe, feasible, and significative staging technique.
Renzi et al. 2008 [54]	22	3	19	64 (35–80)	Face and neck: 15 Upper limbs and trunk: 6 Lower limbs: 1	1/22 (4.5%)	cSCC	SLNB for cSCC is safe and feasible, but its significance is yet to be determined.
Sahn et al. 2007 [55]	9	0	9	66 (52–84)	Face and neck: 4 Upper limbs: 2 Lower limbs: 1 Trunk: 2	0/9 (0.0%)	cSCC	SLNB for cSCC is safe and feasible, but the SLNB results are less predictable compared to melanoma.
Alkureishi et al. 2007 [56]	65	NA	NA	NA	Face and neck: 65	29/65 (44.6%)	Face and neck cSCC	Nodal size is an inaccurate predictor for SLNB results in face and neck cSCC.
Cecchi et al. 2005 [57]	5	NA	NA	NA	NA	1/5 (20.0%)	cSCC (recurrent disease)	SLNB is safe, feasible, and significative in patients with recurrent cSCC.
Eastman et al. 2004 [58]	6	NA	NA	NA	Upper limbs: 4 Lower limbs: 2	4/6 (66.7%)	cSCC (Marjolin’s ulcer)	SLNB is safe, feasible, and significative in patients with Marjolin’s ulcer.
Nouri et al. 2004 [59]	8	0	8	70	Face and neck: 8	1/8 (12.5%)	Face and neck high-risk cSCC	SLNB is a feasible technique, but its significance is yet to be determined.
Wagner et al. 2004 [60]	17	NA	NA	60 (32–93)	Face and neck: 4 Upper limbs: 5 Lower limbs: 3 Vulvar: 5	5/17 (29.4%)	cSCC	SLNB for cSCC is safe and feasible, but SLNB indications are still to be defined.

**Table 2:** Clinical evidence for the role of SLNB in MCC.

Reference	N. Patients	Females	Males	Mean Age	Body Region	SLNB Positivity Rate	Outcomes
Rastrelli et al. 2021 [61]	52	NA	NA	NA	NA	27/52 (51.9%)	SLNB is safe and feasible in MCC. Autoimmune and neoplastic comorbidities are common. Immunomodulatory therapies are considered a negative prognostic factor.
Ahmad et al. 2021 [62]	57	24	33	70 (43–89)	Face and neck: 28 Extremities: 23 Trunk: 6	15/57 (26.3%)	SLNB-negative non-radiotreated patients experienced a 67% of cancer relapse.
Harounian et al. 2021 [63]	76	NA	NA	NA	NA	22/76 (29%)	SLNB and LVI independently correlated to high-risk disease.
Jenkins et al. 2019 [64]	41	13	28	70	Face and neck: 15 Upper Extremities: 5 Trunk: 21	16/41 (39.0%)	SLNB is safe, feasible, and significative in MCC patients.
Conic et al. 2019 [65]	1174	427	747	NA	Face and neck: 406 Extremities: 666 Trunk: 102	361/1174 (30.8%)	Truncal MCC, tumor-infiltrating lymphocytes, and lymphovascular invasion correlate independently to increased risk of positive SLNB.
Sims et al. 2018 [66]	150	45	105	71	Face and neck: 54 Extremities: 81 Trunk: 15	39/150 (26%)	SLNB is safe, feasible, and significative in MCC patients. In-transit recurrence is more common in patients with positive SLNB. These may benefit from adjuvant radiation.
Mattavelli et al. 2017 [67]	64	35	29	69 (31–87)	Face and neck: 13 Upper limbs: 11 Lower limbs: 36 Trunk: 4	17/64 (26.6%)	SLNB is safe and feasible. However, presence of a residual tumor in the specimen of MCC local excision is the main prognostic factor.
Servy et al. 2016 [68]	87	49	38	70 (31–90)	Face and neck: 27 Extremities: 50 Trunk: 10	21/87 (24.1%)	SLNB is safe, feasible, and significative for MCC patients.
Shibayama et al. 2015 [69]	6	NA	NA	NA	NA	1/6 (16.7%)	Positive SLNB correlates with an increased risk of distant metastasis.
Ricard et al. 2015 [70]	12	8	4	74 (62–85)	Face and neck: 12	1/12 (8.3%)	SLNB is safe and feasible, but the significance remains to be defined.
Grotz et al. 2015 [71]	150	NA	NA	NA	NA	39/150 (26.0%)	Regional nodal irradiation can be avoided in SLNB-negative MCC.
Jouary et al. 2015 [72]	108	60	48	70 (21–87)	Face and neck: 30 Upper limbs: 31 Lower limbs: 38 Trunk: 9	33/108 (30.6%)	Immunosuppression and SLNB-positive results are negative prognostic factors for MCC.
Gunaratne et al. 2015 [73]	29	8	21	69 (57–81)	Face and neck: 10 Upper limbs: 10 Lower limbs: 6 Trunk: 3	14/29 (48.3%)	Regional nodal therapy can be avoided in SLNB-negative MCC.
Kachare et al. 2014 [74]	474	189	285	73 (38–99)	NA	115/474 (24.3%)	SLNB is safe, feasible, and significative for MCC patients.
Sattler et al. 2013 [75]	19	5	14	70 (59–85)	Face and neck: 6 Upper limbs: 8 Lower limbs: 3 Trunk: 2	2/19 (10.5%)	SLNB should be indicated in all MCC cases.
Kouzmina et al. 2013 [76]	33	20	13	NA	Face and neck: 13 Upper limbs: 11 Lower limbs: 8 Trunk: 1	10/33 (30.3%)	SLNB is useful and has prognostic value in MCC, regardless of tumor size.
Matthey-Giè et al. 2013 [45]	3	1	2	78 (72–84)	Lower limbs: 3	2/3 (66.7%)	SLNB is safe and feasible, but the impact on survival remains to be defined.
Howle et al. 2012 [77]	16	3	13	64 (37–88)	Face and neck: 8 Upper limbs: 6 Lower limbs: 2 Trunk: 2	8/16 (50.0%)	SLNB is useful and has prognostic value in MCC.
Fields et al. 2011 [78]	153	62	92	69 (60–75)	Face and neck: 33 Extremities: 88 Trunk: 32	45/153 (29.4%)	SLNB is safe and feasible in MCC. Tumor size and presence of lymphovascular invasion correlate to an increased risk of positive SLNB. However, positive SLNB is not associated with recurrence or survival contrary to lymphovascular invasion (strong association with both).
Warner et al. 2008 [79]	11	5	6	74	Face and neck: 7 Upper limbs: 3 Lower limbs: 1	3/11 (27.3%)	SLNB is safe and feasible, but it is not an accurate predictor of locoregional recurrence.
Perez et al. 2007 [80]	8	4	4	64 (34–84)	Upper limbs: 2 Lower limbs: 6	3/8 (37.5%)	SLNB is a safe, feasible, and significative staging technique.
Maza et al. 2006 [81]	23	7	16	70 (50–85)	Face and neck: 3 Upper limbs: 10 Lower limbs: 7 Trunk: 3	11/23 (47.8%)	SLNB is a safe, feasible, and significative staging technique.
Schmalbach et al. 2005 [82]	10	6	4	74 (55–85)	Face and neck: 10	2/10 (20.0%)	SLNB is a safe, feasible, and significative staging technique.
Wagner et al. 2004 [60]	5	NA	NA	69 (65–78)	Upper limbs: 4 Lower limbs: 1	2/5 (40.0%)	SLNB is a safe, feasible, and significative staging technique

**Table 3:** Clinical evidence for the role of SLNB in Porocarcinoma.

Reference	N. Patients	Females	Males	Mean Age	Body Region	SLNB Positivity Rate	Outcomes
Meriläinen et al. 2023 [83]	6	1	5	59 (19–74)	Face and neck: 1 Upper limbs: 1 Lower limbs: 3 Trunk: 1	0/6 (0.0%)	SLNB is safe and feasible, but the indications are still to be defined for Porocarcinoma.
Goyal et al. 2021 [84]	50	NA	NA	NA	NA	18/50 (36.0%)	SLNB has limited prognostic value in patients with advanced malignant cutaneous adnexal carcinoma.
Storino et al. 2021 [85]	4	NA	NA	NA	NA	0/4 (0.0%)	SLNB is safe and feasible, but the impact on survival remains to be defined.
Tsunoda et al. 2019 [17]	8	4	4	69 (59–79)	Face and neck: 1 Upper limbs: 2 Lower limbs: 2 Trunk: 3	3/8 (37.5%)	SLNB is a safe, feasible, and significative staging technique. It should be considered first-line management.
Reina et al. 2018 [86]	2	2	0		Lower limbs: 2	0/2 (0.0%)	SLNB is safe and feasible, but the significance remains to be defined.
Shiohara et al. 2007 [87]	2	0	2	63 (52–75)	Lower limbs: 2	1/2 (50.0%)	SLNB is safe, feasible, and useful.

**Table 4:** Study population and SCC subanalysis.

	Study Population				SCC Subanalysis		
	ALL	SCC	MCC	Porocarcinoma	cSCC	cSCC—Face and Neck	Anogenital SCC
N. studies	70	42	24	6	26	10	16
N. patients	6379	3546 55.6% (3546/6379)	2761 43.3% (2761/6379)	72 0.8% (72/6379)	1143 32.2% (1143/3546)	540 15.2% (540/3546)	2403 67.9% (2406/3546)
Females	45.5%	50.1%	39.3%	38.9%	24.8%	22.7%	60.3%
Males	54.5%	49.9%	60.7%	61.1%	75.2%	77.3%	39.7%
Mean Age/mean age range	65 (59–74)	62 (59–73)	70 (69–73)	63 (59–69)	65 (60–74)	77 (60–73)	63 (59–67)
Face and neck	33.7%	24.7%	61.5%	11.1%	88.1%	100%	0%
Upper limb	3.3%	1.4%	8.9%	16.7%	4.5%	0%	0%
Lower Limb	4.0%	1.8%	10.2%	50%	5.8%	0%	0%
Trunk	5.1%	0.4%	19.4%	22.2%	1.6%	0%	0%
Vulvar	30.0%	39.9%	0%	0%	0%	0%	55.6% (1337/2403)
Penile	19.2%	25.5%	0%	0%	0%	0%	35.8% (859/2403)
Anal–perianal	4.8%	6.3%	0%	0%	0%	0%	8.6% (207/2403)
SLNB detection rate	97.6% (4930/5050)	96.4% (2841/2946)	99.3% (2074/2089)	100% (18/18)	93.1% (892/958)	97.9% (465/475)	98.0% (1949/1985)
SLNB positivity rate	24.4% (1557/6379)	20.5% (726/3546)	29.3% (809/2761)	30.6% (22/72)	12.3% (141/1143)	15.9% (86/540)	24.4% (585/2403)
SLNB is safe, feasible, and significative (n. studies)	90.3% (63/70)	88.1% (37/42)	96.8% (23/24)	83.3% (5/6)	80.8% (21/26)	100% (10/10)	100% (16/16)
SLNB is poorly significative (n. studies)	10.0% (7/70)	11.9% (5/42)	4.2% (1/24)	16.7% (1/6)	29.4% (5/26)	0% (0/10)	0% (0/16)

**Table 5:** Criteria for high-risk SCC. ✓ indicates the parameters considered as risk factors.

SCC High-Risk Features	NCCN	AJCC	BWH
Size ≥ 20 mm	✓	✓	✓
Tumor with poor differentiation	✓	✓	✓
Perineural invasion	✓	✓	✓
Bone invasion	✓	✓	✓
2 mm or greater in tumor depth	✓	✓	
Anatomic region: ear, lip (non hair-bairing)	✓	✓	
Anatomic region: scalp, cheek, forehead, neck (≥10 mm)	✓		
Anatomic region: central face, eyelids, periorbital, periauricular, nose temple (≥6 mm)	✓		
Immunosuppression	✓		
Fast-growing tumor	✓		
Recurrent tumor	✓		
Presence of neurologic symptoms	✓		
Previous local radiotherapy or choric inflammatory process	✓		
Adenosquamous, desmoplastic, or acantholytic hystotipes	✓		
Extension beyond subcutaneous fat			✓

## Data Availability

The original contributions presented in the study are included in the article; further inquiries can be directed to the corresponding author.
